# From Model Plants to Staple Crops: Molecular Mechanisms of Plant Saline–Alkali Tolerance

**DOI:** 10.3390/plants15040532

**Published:** 2026-02-08

**Authors:** Delong Fan, Jing Ruan, Qinan Xu, Jiezheng Ying, Yifeng Wang, Xiaohong Tong, Zhiyong Li, Yu Cheng, Dawei Xue, Jian Zhang, Jie Huang

**Affiliations:** 1 College of Life and Environmental Sciences, Hangzhou Normal University, Hangzhou 311121, China; fdllytc@163.com (D.F.); jingr627@163.com (J.R.); xuqinan0119@163.com (Q.X.); 2 State Key Laboratory of Rice Biology and Breeding, China National Rice Research Institute, Hangzhou 311400, China; yingjiezheng@caas.cn (J.Y.); wangyifeng@caas.cn (Y.W.); tongxiaohong@caas.cn (X.T.); lizhiyong@caas.cn (Z.L.); chengyu@caas.cn (Y.C.); 3 National Nanfan Research Institute (Sanya), Chinese Academy of Agricultural Sciences, Sanya 572024, China

**Keywords:** saline–alkali stress, model plant, staple crops, SOS signaling pathway, molecular breeding

## Abstract

Soil salinization, as a key constraint to global agricultural sustainable development, has threatened over one billion hectares of farmland, posing severe challenges to staple crop production. Therefore, this review summarizes important advances in the molecular mechanisms of salt–alkali tolerance from the model plant *Arabidopsis thaliana* to staple crops (rice, maize, and wheat) and compares the commonalities and differences in physiological structure and molecular regulatory networks among these species. Studies have shown that plants respond to saline–alkali stress mainly through conserved mechanisms, including salt overly sensitive (SOS) signaling pathway-mediated ion homeostasis, accumulation of osmoprotectants, reactive oxygen species (ROS) scavenging, and coordination of multiple hormone signals. However, different species have evolved unique adaptive strategies: Arabidopsis has revealed core regulatory pathways, but its simple root system limits direct application in crops; rice employs root barriers and a stem node “ion filter” to precisely regulate Na^+^ transport; maize utilizes the C4 photosynthetic pathway along with efficient osmotic adjustment and tissue compartmentalization to enhance tolerance; and wheat achieves ion detoxification through *TaHKT* allele variation and vacuolar sequestration. Looking forward, future breeding for salt–alkali tolerance should adopt a “crop-centric” approach, focusing on the mining and molecular design of superior alleles, combined with gene editing and multi-trait integration, to provide a theoretical basis and strategic support for developing high-yield and stable crop varieties adapted to saline–alkali lands.

## 1. Introduction

Soil salinization is one of the most severe abiotic stresses facing global agricultural production and ecological security [1]. It is primarily manifested in three stress types: salt stress (mainly caused by neutral salts, which inhibit plant growth through high osmotic pressure and ion toxicity), alkali stress (primarily induced by alkaline salts, which damage root systems via high pH and trigger nutrient deficiencies), and the more complex and severe saline–alkali stress (involving both neutral and alkaline salts, characterized by multiple synergistic hazards including high osmotic pressure, ion toxicity, strong alkalinity, and soil compaction). It is estimated that over one billion hectares of land worldwide are affected by salinization, and this number continues to grow, posing a significant threat to global food security and sustainable agricultural development [2]. Against this backdrop, gaining a deeper understanding of the molecular mechanisms of plant saline–alkali tolerance and utilizing modern molecular breeding techniques to develop new crop varieties adapted to saline–alkaline environments have become major strategic demands in the fields of agriculture and life sciences.

During long-term evolution, plants have developed a series of complex and sophisticated mechanisms to cope with saline–alkali stress [3]. These mechanisms encompass multiple levels, including the regulation of ion homeostasis (such as SOS signaling pathway-mediated Na^+^ efflux and vacuolar sequestration), osmotic balance adjustment (e.g., the accumulation of compatible solutes like proline and glycine betaine), the activation of ROS scavenging systems, and the integration and reprogramming of plant hormone signaling networks (such as ABA and ethylene). A profound understanding of these fundamental physiological and molecular processes forms the theoretical foundation for conducting saline–alkali tolerance breeding.

Research on model plants, represented by *Arabidopsis thaliana* (*A. thaliana*), has made irreplaceable contributions to our understanding of the “core toolkit” for plant saline–alkali tolerance [4]. Its clear genetic background, mature transgenic systems, and rich mutant libraries have enabled scientists to systematically elucidate the core roles of key components in stress response at the molecular level, such as the SOS pathway, NHX transporters, HKT regulators, and key transcription factors like DREB/CBF and NAC [5,6,7,8,9,10]. However, as a dicotyledonous short-cycle herbaceous plant, *A. thaliana* has a simple root system architecture, lacks a long-distance ion transport and filtration system, and exhibits significant differences in physiological responses compared to monocotyledonous staple crops such as rice (*Oryza sativa* L.), maize (*Zea mays* L.), and wheat (*Triticum aestivum* L.) [11]. Consequently, directly applying research findings from model plants to crop improvement faces numerous challenges, creating an urgent need to dissect the mechanisms and validate gene functions within the specific genetic contexts of the crops themselves [12].

As the three major staple crops globally, rice, maize, and wheat have evolved unique adaptation strategies to cope with saline–alkali stress. For instance, rice relies on its well-developed root barrier and the “ion filter” function of its stem nodes to finely regulate the allocation of Na^+^ to the shoots, particularly the panicles [13]. Maize benefits from its highly efficient C4 photosynthetic pathway, which maintains relatively stable carbon assimilation efficiency under stress, coupled with strong osmotic adjustment and tissue-level ion compartmentalization capabilities [14]. Wheat, through complex allelic variations in *TaHKT* genes and efficient vacuolar sequestration mechanisms, achieves ion redistribution and detoxification [15]. The existence of these species-specific mechanisms implies that future breeding strategies must shift from a “model-oriented” to a “crop-centric” approach, deeply mining and utilizing the superior alleles present in crop germplasm resources [16].

This review aims to systematically summarize recent research advances in the molecular mechanisms of saline–alkali tolerance, spanning from the model plant *A. thaliana* to the staple crops rice, maize, and wheat. The article will focus on comparing the commonalities and specificities among different species in areas such as ion homeostasis, osmotic regulation, antioxidant defense, and signal transduction networks. It will also summarize the unique physiological and structural adaptations they have evolved. Finally, this paper will provide perspectives on future research directions and breeding strategies, with the goal of offering a solid theoretical foundation and feasible technical pathways for advancing the genetic improvement of saline–alkali tolerant crops and the effective utilization of saline–alkali soil resources.

## 2. The Molecular Mechanisms of Saline–Alkali Stress Tolerance in *A. thaliana*

As a model organism in plant biology research, *A. thaliana* has become the preferred material for studying plant saline–alkali tolerance mechanisms, owing to its advantages such as a small genome (approximately 135 Mb), short life cycle (6–8 weeks), high genetic transformation efficiency (can exceed 80%), and extensive T-DNA insertion mutant libraries [17,18]. During long-term evolution, *A. thaliana* has developed a comprehensive regulatory network for saline–alkali tolerance, spanning from signal perception to systemic responses.

**Figure 1:**
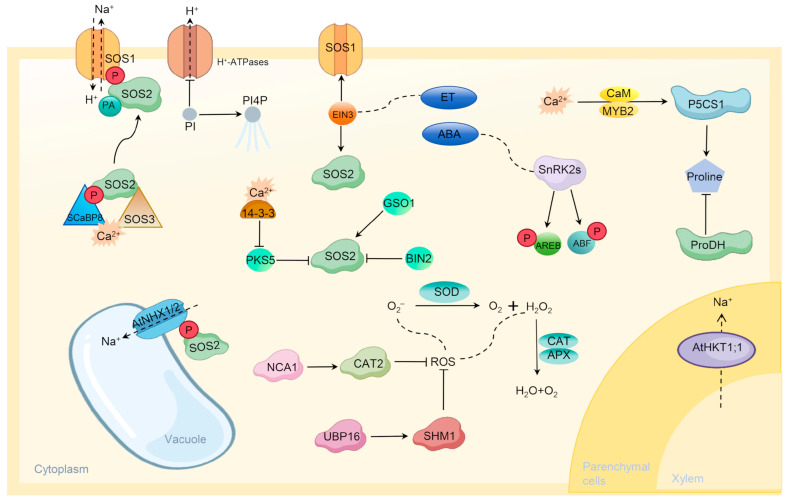
Research advances in *A. thaliana* tolerance to saline–alkali stress. Under saline–alkali stress, cytosolic Ca^2+^ levels rapidly increase and are perceived by calcium-binding proteins SOS3 and SCaBP8. These sensor proteins activate the protein kinase SOS2, which in turn phosphorylates and activates the plasma membrane Na^+^/H^+^ antiporter SOS1. SOS1 utilizes the proton gradient established by the plasma membrane H^+^-ATPase to actively pump Na^+^ out of the cell, directly reducing cytosolic Na^+^ concentration. This core SOS pathway is also regulated by phospholipid signaling molecules (PI, PI4P, and PA). The vacuolar membrane Na^+^/H^+^ antiporters AtNHX1 and AtNHX2 pump Na^+^ from the cytoplasm into the vacuole. The high-affinity potassium transporter AtHKT1;1 facilitates the recirculation of Na^+^ from the xylem to the phloem by loading Na^+^ from the xylem sap into surrounding parenchyma cells. Saline–alkali stress strongly induces the expression of *P5CS1*, a key gene in proline synthesis while suppressing the expression of the degradation gene *ProDH*, thereby enhancing *A. thaliana*’s tolerance to salt and osmotic stress. The massive accumulation of reactive oxygen species (ROS) activates its antioxidant system, which includes increasing the activity of antioxidant enzymes (such as CAT2) and synthesizing antioxidant compounds, to scavenge ROS and protect cells from oxidative damage. The ubiquitin-specific protease UBP16 stabilizes serine hydroxymethyltransferase SHM1 through deubiquitination, thereby indirectly regulating ROS levels. Abscisic acid (ABA) levels rise rapidly under salt stress. Activated SnRK2s kinases phosphorylate downstream transcription factors such as AREB/ABF, which subsequently activate the expression of numerous stress-responsive genes and regulate stomatal closure to reduce water loss. The ethylene signaling pathway is closely linked to saline–alkali tolerance. Its key transcription factor EIN3 can directly activate the expression of *SOS1* and *SOS2*, establishing a direct bridge between hormone signaling and ion homeostasis regulation.

Among these, the SOS pathway is the most thoroughly studied core signaling pathway [19]. This pathway is initiated by the increase in cytosolic Ca^2+^ signals triggered by salt stress. The calcium sensor protein SOS3 and its homolog SCaBP8 perceive the elevated Ca^2+^ and interact with the serine/threonine protein kinase SOS2, relieving its autoinhibition and thereby activating SOS2 [20,21]. The activated SOS2 subsequently phosphorylates and activates the plasma membrane-localized Na^+^/H^+^ antiporter SOS1, which utilizes the proton gradient established by the plasma membrane H^+^-ATPase to actively pump Na^+^ out of the cell, directly reducing the cytosolic Na^+^ concentration [22]. In addition to this plasma membrane efflux mechanism, sequestering excess Na^+^ into the vacuole represents another crucial detoxification strategy. This process is primarily carried out by vacuolar membrane Na^+^/H^+^ antiporters, such as *AtNHX1* and *AtNHX2*, which utilize the inward H^+^ gradient generated by the vacuolar membrane H^+^-ATPase and H^+^-PPase to pump cytosolic Na^+^ into the vacuole [6]. It is noteworthy that the SOS2 kinase can also phosphorylate and activate NHX transporters, indicating that the plasma membrane efflux and vacuolar sequestration processes are coordinately regulated through signaling pathways (Figure 1) [23]. Regarding the restriction of Na^+^ uptake, the high-affinity potassium transporter AtHKT1;1 in *A. thaliana* plays a unique role. It is primarily expressed in the pericycle and xylem parenchyma cells, where it facilitates the recirculation of Na^+^ from the xylem to the phloem by loading Na^+^ from the xylem sap into the surrounding parenchyma cells. This process effectively reduces Na^+^ transport to the aerial leaves, thereby protecting the photosynthetic organs [7,24].

Beyond ionic toxicity, osmotic stress and oxidative stress are also major components of salt damage. In terms of osmotic adjustment, plants reduce cellular osmotic potential by synthesizing and accumulating compatible solutes. In *A. thaliana*, proline accumulation serves as a classic example; salt stress strongly induces the expression of *P5CS1*, a key gene in its biosynthesis, while repressing the degradation gene *ProDH*. This process is precisely regulated by various transcription factors (Figure 1) [25]. Biosynthetic pathways for solutes like glycine betaine and trehalose have also been confirmed to contribute to osmotic protection [26]. In coping with oxidative stress, *A. thaliana* possesses a complex ROS scavenging network. The enzymatic system includes superoxide dismutase (SOD), ascorbate peroxidase (APX), and catalase (CAT), among others. The high salt sensitivity of the cytosolic *APX1* mutant underscores its critical role. The non-enzymatic system relies on antioxidants such as ascorbic acid (ASA) and glutathione (GSH) [27,28].

Plant hormones are core signaling molecules regulating the salt stress response, forming a complex integrative network. Abscisic acid (ABA), the primary stress hormone, rapidly accumulates under salt stress and transmits signals via the PYR/PYL/RCAR receptor-PP2C-SnRK2 core signaling module. Activated SnRK2s kinases phosphorylate downstream transcription factors like AREB/ABF, thereby activating a multitude of stress-responsive genes and regulating stomatal closure to reduce water loss [29,30]. The ethylene signaling pathway is closely linked to salt tolerance, as its key transcription factor EIN3 can directly activate the expression of SOS1 and SOS2, establishing a direct bridge between hormone signaling and ion homeostasis regulation (Figure 1) [31]. Furthermore, hormone signals such as brassinosteroids (BR), jasmonic acid (JA), and salicylic acid (SA) engage in extensive crosstalk with stress signaling pathways through their core components, finely balancing plant growth and defense responses [32].

In response to alkaline stress, plants have evolved specific adaptation mechanisms distinct from those for salt stress. The high-pH environment caused by alkaline stress poses a severe challenge to cellular pH homeostasis. Plants activate specific signaling pathways to counter this stress. The calcium sensor SCaBP3 dissociates from the plasma membrane H^+^-ATPase AHA2 under alkaline stress, relieving the inhibition of its proton pump activity and promoting H^+^ efflux to lower the apoplastic pH (Figure 1). The DnaJ-like molecular chaperone J3 indirectly enhances H^+^-ATPase function by inhibiting PKS5 kinase activity, working cooperatively to maintain pH homeostasis [33]. Additionally, the sorghum AT1 gene influences H_2_O_2_ efflux by modulating the phosphorylation status of the aquaporin PIP2;1, and loss of its function can enhance plant alkaline tolerance [34].

## 3. The Molecular Mechanisms of Saline–Alkali Stress Tolerance in Rice

**Figure 2:**
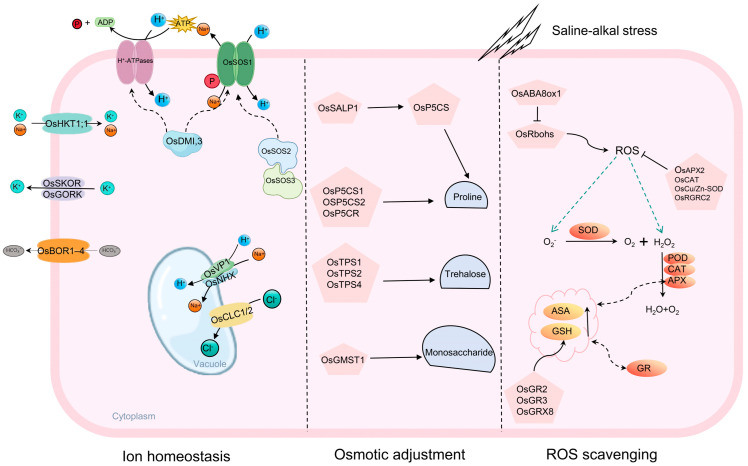
Research advances in rice tolerance to saline–alkali stress. In rice, the exclusion of Na^+^ from shoots is regulated by the Na^+^/H^+^ antiporter OsSOS1. OsDMI3 enhances salt tolerance during root growth by modulating OsSOS1 and H^+^-ATPase under salt stress. The transporter OsHKT1;1 contributes to reduced Na^+^ accumulation in leaves, while the shaker-like potassium channels OsSKOR and OsGORK regulate K^+^ homeostasis. The vacuolar Na^+^/H^+^ antiporter OsNHX sequesters excess cytosolic Na^+^ into the vacuole, thereby enhancing tolerance to saline–alkali stress. OsVP1 also facilitates Na^+^/H^+^ exchange and improves rice salt resistance by pumping H^+^ from the cytoplasm into the vacuole. The chloride channel proteins OsCLC1 and OsCLC2 mediate Cl^−^ transport into the vacuole, bolstering plant salt tolerance and normal development. The HCO_3_^−^ transporter encoded by the *SLC4* family gene helps maintain cytosolic pH homeostasis by extruding HCO_3_^−^. The upregulated expression of proline biosynthesis genes *OsP5CS1*, *OsP5CS2*, and *OsP5CR* promotes proline accumulation, enhancing rice tolerance to both saline–alkali and osmotic stress. Similarly, trehalose synthesis genes *OsTPS1*, *OsTPS2*, and *OsTPS4* increase trehalose content, thereby improving resistance to high salt, high pH, and drought stress. Under saline–alkali stress, *OsABA8ox1* reduces ROS accumulation and scavenges ROS produced by *OsRbohs*, conferring tolerance in rice plants. Superoxide dismutase (SOD), the first enzyme in the antioxidant system, converts accumulated O_2_^−^ into O_2_ and H_2_O_2_. Subsequently, POD, CAT, and APX decompose H_2_O_2_ into H_2_O and O_2_.

Saline–alkali stress severely constrains the growth and development of rice, with its primary damage mechanisms including ionic toxicity, osmotic stress, and oxidative damage. To cope with this adversity, rice has evolved multi-layered physiological response mechanisms, specifically manifested in processes such as ion homeostasis regulation, osmotic balance maintenance, and ROS scavenging [35]. Regarding ion homeostasis, maintaining intracellular Na^+^/K^+^ balance is crucial for rice’s saline–alkali tolerance. Since Na^+^ and K^+^ share transport systems, rice employs strategies such as limiting Na^+^ uptake, enhancing efflux, and vacuolar sequestration to alleviate Na^+^ toxicity under saline–alkali stress [36]. For instance, the plasma membrane Na^+^/H^+^ antiporter OsSOS1 mediates Na^+^ efflux from cells, while vacuolar membrane Na^+^/H^+^ antiporters like OsNHX1 sequester excess Na^+^ into the vacuole, thereby maintaining a low cytosolic Na^+^/K^+^ ratio [37,38]. Furthermore, members of the high-affinity K^+^ transporter family, such as OsHKT1;1 and SKC1, effectively reduce Na^+^ accumulation in leaves [39]. Chloride channel proteins OsCLC1 and OsCLC2 enhance plant salt tolerance and normal development by mediating Cl^−^ transport into the vacuoles [40]. HCO_3_^−^ transporters encoded by SLC4 family genes help maintain cytosolic pH homeostasis by extruding HCO_3_^−^ (Figure 2) [40].

In terms of osmotic adjustment, saline–alkali stress increases osmotic potential, inducing rice to accumulate soluble osmolytes such as sucrose, proline, glycine betaine, and polyamines to lower cellular osmotic potential and stabilize protein structures and membrane systems [26]. Upregulated expression of proline biosynthesis-related genes *OsP5CS1*, *OsP5CS2*, and *OsP5CR* promotes proline accumulation, enhancing rice tolerance to saline–alkali and osmotic stress [41]. Trehalose synthesis genes *OsTPS1*, *OsTPS2*, and *OsTPS4* improve resistance to high salt, high pH, and drought stress by increasing trehalose content (Figure 2) [42]. The synthesis of some secondary metabolites, like diterpenoids and phenylpropanoids, has also been shown to participate in the saline–alkali stress response [43]. Concurrently, increased activities of antioxidant enzymes such as SOD, CAT, and POD in the roots help mitigate damage induced by high-pH alkaline stress [44]. Concerning the ROS scavenging mechanism, ion imbalance and osmotic disturbance caused by saline–alkali stress lead to ROS accumulation, which disrupts cellular metabolism and damages biological macromolecules. Rice mitigates ROS toxicity through enzymatic and non-enzymatic antioxidant systems: the enzymatic system includes respiratory burst oxidase homologs (RBOHs), SOD, and APX; the non-enzymatic system relies on antioxidants like ascorbic acid, secondary metabolites, and carotenoids [28]. Additionally, rice enhances its saline–alkali tolerance by maintaining nutrient balance, regulating intracellular pH homeostasis, and ensuring normal nitrogen metabolism.

At the molecular level, saline–alkali stress activates various signaling molecules, including phospholipids, hormones, and Ca^2+^, which subsequently initiate downstream response networks to maintain ion homeostasis and osmotic balance, thereby alleviating growth inhibition. For example, the Ca^2+^/calmodulin-dependent protein kinase gene *OsDMI3* coordinates Na^+^ and H^+^ transmembrane transport by regulating *OsSOS1* and the plasma membrane H^+^-ATPase genes *OsHA3*/*OsHA8*, promoting root growth under saline–alkali stress (Figure 2) [45]. Overexpression of *OsCam1-1* induces ABA accumulation, thereby improving salt tolerance; conversely, inhibiting the key ABA catabolism gene *OsABA8ox1* helps scavenge plasma membrane peroxides and enhances salt tolerance (Figure 2) [46,47]. To date, over 40 transcription factors involved in the saline–alkali stress response have been identified, spanning families such as AP2/ERF, bZIP/HD-Zip, MYB/MYC, WRKY, and NAC. These factors play a central regulatory role in stress signal transduction by recognizing and binding to cis-acting elements in downstream target genes [44,48].

## 4. The Molecular Mechanisms of Saline–Alkali Stress Tolerance in Maize

The saline–alkali tolerance of maize is a complex quantitative trait controlled by multiple genes, and elucidating its molecular mechanisms is crucial for breeding new maize varieties with enhanced tolerance. In recent years, the integrated application of multi-omics technologies—such as genomics, transcriptomics, proteomics, and metabolomics—has greatly advanced this field, leading to a series of important discoveries in ion homeostasis regulation, osmotic balance, oxidative stress response, and nutrient uptake [49].

**Figure 3:**
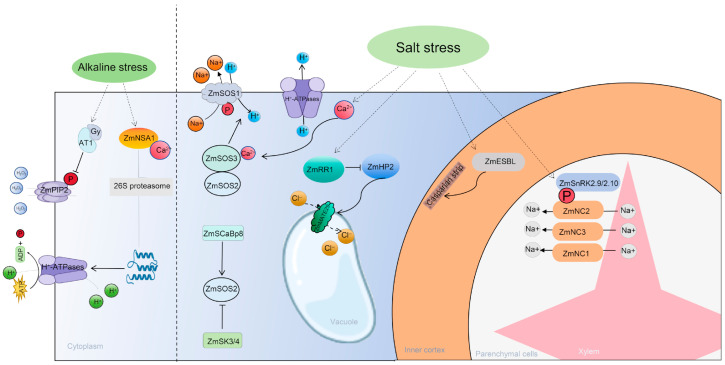
Research advances in maize tolerance to saline–alkali stress. Salt stress triggers an increase in cytosolic Ca^2+^ concentration, which activates the SOS signaling pathway and consequently enhances ZmSOS1-mediated root Na^+^ efflux. Salt stress promotes the degradation of ZmRR1, alleviating its inhibitory effect on *ZmHP2*. The enhanced cytokinin signaling pathway mediated by *ZmHP2* upregulates the transcription of *ZmMATE29*, facilitating Cl^−^ sequestration into the vacuoles of root cortical cells. This process reduces Cl^−^ accumulation in the shoots and improves salt tolerance in maize. Salt stress leads to increased transcription of *ZmESBL*, which promotes the development of the Casparian strip and strengthens its barrier function. This enhanced barrier prevents excessive Na^+^ from entering the root stele via the apoplastic pathway while promoting Na^+^ exclusion from the shoots, thereby improving maize salt tolerance. Na^+^-selective transporters (ZmNC1, ZmNC2, ZmNC3, and ZmHAK11) unload Na^+^ from the xylem into surrounding parenchyma cells, thereby enhancing shoot Na^+^ exclusion and salt tolerance in maize. Under alkaline stress, AT1 pairs with the G-protein β subunit, reducing the phosphorylation of the plasma membrane aquaporin PIP2 and decreasing its H_2_O_2_ efflux activity. This leads to elevated intracellular ROS levels, resulting in a severely alkaline-sensitive phenotype. Concurrently, Ca^2+^ binds to ZmNSA1 and induces its degradation via the 26S proteasome pathway. The degradation of ZmNSA1 subsequently increases the transcription levels of MHAs (plasma membrane H^+^-ATPases), promoting H^+^ efflux.

The SOS signaling pathway in maize has now been fully elucidated: under salt stress conditions, elevated cytosolic Ca^2+^ levels are perceived by the ZmSOS3 protein. ZmSOS3 then binds to and activates ZmSOS2. The activated ZmSOS3-ZmSOS2 complex subsequently phosphorylates ZmSOS1, thereby activating its Na^+^ transport activity to promote Na^+^ efflux (Figure 3) [50]. Furthermore, this pathway is finely modulated by negative regulators like ZmSK3/ZmSK4 and positive regulators like ZmSCaBP8 [51]. In addition to root efflux, xylem Na^+^ unloading is a key process limiting Na^+^ transport to the shoots. Genes such as ZmHKT1;1, ZmHKT1;2, and ZmHAK4, expressed in the stele parenchyma cells, are responsible for transferring Na^+^ from the xylem to surrounding cells (Figure 3) [52,53,54]. Moreover, the root endodermal Casparian strip acts as an apoplastic barrier, playing a vital role in restricting Na^+^ influx. The *ZmSTL1*/*ZmESBL* gene encodes a DIR family protein that positively regulates Casparian strip development [55]. Its upregulated expression under salt stress enhances the barrier function of the Casparian strip, effectively preventing Na^+^ from entering the stele via the apoplastic pathway. Loss of function of this gene leads to a sharp increase in shoot Na^+^ content, indicating that the developmental plasticity of the Casparian strip is an important mechanism for maize adaptation to salt stress. Beyond Na^+^, the homeostatic balance of K^+^ and Cl^−^ is equally critical. *ZmHKT2* has been identified as a QTL negatively regulating K^+^ content in shoots; loss of its function conversely enhances salt tolerance [56]. Regarding Cl^−^ regulation, ZmRR1 upregulates the expression of the vacuolar membrane Cl^−^ transporter ZmMATE29 by suppressing the cytokinin signaling pathway, promoting Cl^−^ sequestration into root cortical vacuoles and thereby enhancing salt tolerance (Figure 3) [57]. A non-synonymous polymorphism, SNP307-T, impairs this pathway, resulting in reduced salt tolerance.

In response to alkaline stress, *ZmNSA1* encodes an EF-hand calcium-binding protein that affects root proton efflux and Na^+^ extrusion by negatively regulating the expression of plasma membrane H^+^-ATPases [58]. Additionally, the Gγ protein SbAT1 (and its maize ortholog) has been shown to negatively regulate alkaline stress tolerance by influencing the phosphorylation of the aquaporin PIP2 and H_2_O_2_ efflux; its knockout significantly improves maize survival rate (Figure 3) [59]. Meanwhile, other mechanisms collectively constitute the saline–alkali tolerance network in maize. Metabolomic analyses have identified several marker metabolites associated with osmotic stress resistance and revealed that functional variations in genes such as *ZmCS3*, *ZmUGT*, and *ZmCYP709B2* correlate with salt tolerance [60]. Overexpression of the glycine betaine synthesis-related gene *ZmGB1* and the raffinose synthesis gene *ZmGolS1* both enhance salt tolerance [61,62]. At the signal transduction level, *ZmMPK3* can phosphorylate and stabilize the transcription factor ZmGRF1, forming a signaling module that regulates saline–alkali tolerance [63].

## 5. The Molecular Mechanisms of Saline–Alkali Stress Tolerance in Wheat

**Figure 4:**
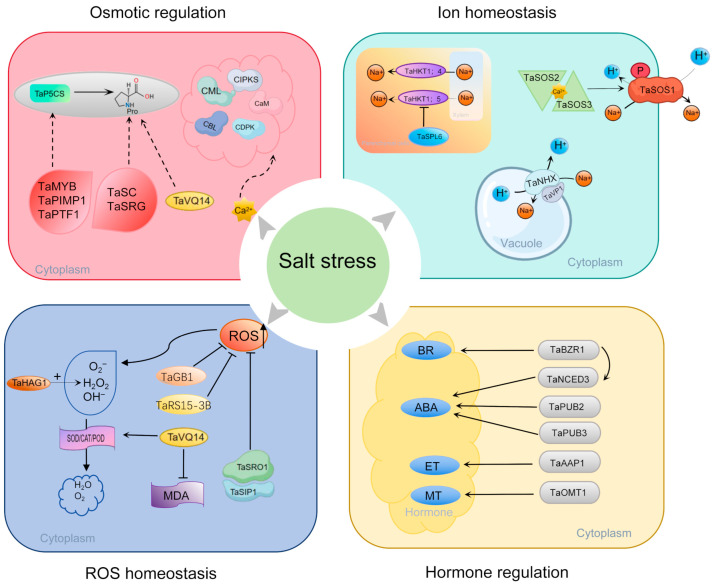
Research advances in wheat tolerance to saline–alkali stress. Under saline–alkali stress, the rapid increase in salt concentration around wheat roots induces osmotic stress, leading to the activation of hyperosmosensors. This triggers calcium ion signaling and initiates a series of responses involving *CDPKs*, *CBLs*, and *CIPKs*. The upregulated expression of proline biosynthesis-related genes such as *TaMYB*, *TaPIMP1*, *TaPTF1*, and *TaSC* promotes proline accumulation, enhancing wheat tolerance to saline–alkali and osmotic stress. Ca^2+^ binds to SOS3 and SOS2, activating SOS1 through phosphorylation. The activated SOS1 facilitates the efflux of Na^+^ into the xylem apoplast or soil. The NHX (Na^+^/H^+^ antiporter) family plays a critical role in sequestering Na^+^ into vacuoles. Furthermore, under saline–alkali stress, the key agronomic trait-related factor TaSRO1 in wheat enhances ROS scavenging capacity. Antioxidant enzymes such as SOD, CAT, and POD are utilized to eliminate excess reactive oxygen species and mitigate oxidative stress. Hormones, including ABA, ET, BR, and MT, also play regulatory roles in the response to saline–alkali stress.

As one of the world’s most important cereal crops, the stable production of wheat is crucial for ensuring global food security. However, saline–alkali stress severely restricts its cultivation area and production potential. To address this challenge, wheat has evolved a series of complex physiological and molecular adaptation mechanisms during its long-term evolution. Its saline–alkali tolerance is a complex quantitative trait controlled by multiple genes, involving the synergistic action of multiple layers, including the accumulation of osmotic adjustment compounds, regulation of ion homeostasis, scavenging of reactive oxygen species (ROS), and hormonal signaling networks. In terms of osmotic adjustment, wheat initiates the primary stress response by activating the calcium signaling system, which includes proteins such as CaM, CML, CDPK, CBL, and CIPK. Studies have shown that overexpression of *TaCAM2-D* significantly enhances dual tolerance to both drought and saline–alkali stress, whereas silencing *TaCDPK27* leads to impaired root development and increased salt sensitivity in wheat seedlings [64]. Concurrently, wheat maintains cell turgor by accumulating osmolytes like proline. The expression of *TaP5CS*, a key gene in proline biosynthesis, is significantly upregulated under saline–alkali stress. This process is finely regulated by various transcription factors, including TaMYB, TaPIMP1, and TaPTF1, forming a complex osmotic protection network (Figure 4) [65,66,67]. Saline–alkali tolerance-related genes *TaSC* and *TaSRG* play key roles in osmotic adjustment by regulating the rate of proline biosynthesis.

In maintaining ion homeostasis, wheat employs multiple coordinated pathways to regulate Na^+^ transport and compartmentalization. The high-affinity potassium transporters TaHKT1;5 and TaHKT1;4 transfer Na^+^ from the xylem to surrounding parenchyma cells, effectively reducing Na^+^ transport to the shoots [15,68]. This process is finely regulated by the transcription factor TaSPL6. The allelic variant *TaSPL6-DIn*, which contains an insertion mutation, loses its inhibitory effect on TaHKT1;5, thereby enhancing plant saline–alkali tolerance (Figure 4) [69,70]. The classic SOS signaling pathway plays a central role in plasma membrane Na^+^ efflux. *TaSOS1* has been confirmed to possess Na^+^/H^+^ exchange activity in yeast and transgenic tobacco, and it improves saline–alkali tolerance [71]. Furthermore, the vacuolar membrane Na^+^/H^+^ antiporter family TaNHX, in coordination with the proton pump TaVP1, sequesters excess Na^+^ into the vacuole, effectively alleviating cytosolic Na^+^ toxicity [72]. Research indicates that coexpression of *TaNHX1* and *TaVP1* significantly enhances saline–alkali tolerance in transgenic plants.

Regarding ROS balance, wheat relies on both enzymatic and non-enzymatic antioxidant systems to maintain redox homeostasis. Key antioxidant enzymes include SOD, CAT, and POD. *TaVQ14* effectively reduces oxidative damage by increasing the activities of CAT and SOD, raising proline content, and simultaneously decreasing malondialdehyde (MDA) levels (Figure 4) [73]. Genes such as *TaGB1* and *TaRS15-3B* also enhance saline–alkali tolerance by boosting the functionality of the antioxidant system. It is noteworthy that ROS plays a dual role in plant stress responses: excessive accumulation causes oxidative damage, whereas moderate accumulation acts as a signaling molecule to activate defense mechanisms [74,75]. The histone acetyltransferase TaHAG1 positively regulates saline–alkali tolerance by modulating the transcription of genes related to H_2_O_2_ production [76]. Meanwhile, the TaSRO1-TaSIP1 protein interaction module ensures the stress response is not overactivated through precise negative regulation of ROS signaling; the balance of this module is crucial for plant survival (Figure 4) [77].

Plant hormones act as central hubs in integrating saline–alkali stress signals and regulating the global response. ABA, a key stress hormone, has its signal transduction mediated by E3 ubiquitin ligases such as TaPUB2 and TaPUB3 [78]. In the ethylene biosynthesis pathway, *TaAAP1* enhances ethylene levels, improving saline–alkali tolerance during wheat germination and the seedling stage [79]. The core transcription factor TaBZR1 in the brassinosteroid signaling pathway directly activates *TaNCED3*, a key gene in ABA synthesis, as well as ROS-scavenging genes *TaGPX2* and *TaGPX3*, thereby mediating the crosstalk of hormone signals and synergistic enhancement of antioxidant defense [80]. Additionally, the melatonin synthesis gene *TaOMT1* enhances the antioxidant system by elevating endogenous melatonin levels, playing a significant role in re-establishing redox homeostasis (Figure 4) [81]. These findings provide a new theoretical basis for in-depth analysis of the molecular mechanisms of saline–alkali tolerance in wheat and lay an important foundation for the molecular breeding of saline–alkali-tolerant wheat varieties.

## 6. Comparative Analysis of Molecular Mechanisms in Plant Response to Saline–Alkali Stress

Saline–alkali stress is one of the major abiotic stressors facing global agricultural production [19]. Understanding plant tolerance mechanisms is crucial for breeding stress-resistant crop varieties. Research using *A. thaliana* as a model plant has provided a fundamental theoretical framework and candidate gene resources for plant biology [5]. However, applying findings from this model plant to staple crop breeding requires careful consideration of significant differences in physiological structure, photosynthetic pathways, and ecological adaptation [82].

Ion homeostasis maintenance is a fundamental survival mechanism for all plants. This process is typically achieved through SOS pathway-mediated intracellular Na^+^ efflux and vacuolar membrane NHX-type Na^+^/H^+^ antiporter-mediated vacuolar sequestration (Table 1) [83]. *A. thaliana* has a relatively simple root system architecture, and its saline–alkali tolerance primarily relies on cellular-level regulation, with overall root salt exclusion capacity being relatively weak [84]. In contrast, cereal crops have evolved more complex regulatory strategies involving root and vascular systems. As a salt-sensitive crop, rice’s saline–alkali tolerance largely depends on root salt exclusion capacity and selective Na^+^ transport in stem nodes [85]. Tolerant rice varieties effectively limit initial Na^+^ uptake through outer root cells, and their stem nodes function as efficient “ion filters.” Transporters like OsHKT1;5 reload Na^+^ from the xylem sap into surrounding parenchyma cells, sequestering it in lower leaf sheaths and older leaves, thereby minimizing Na^+^ transport to photosynthetically active new leaves and reproductive organs (Table 1) [86,87]. Maize and wheat, also belonging to the Poaceae family, employ a different strategies: both exhibit moderate root salt exclusion capacity but emphasize tissue-level ion compartmentalization, effectively isolating absorbed Na^+^ in older leaves as an adaptive strategy of sacrificing older leaves to protect new ones [88]. Additionally, maize maintains a high K^+^/Na^+^ ratio through an efficient K^+^ uptake system, forming an important physiological basis for its tolerance, while tolerant wheat varieties retain Na^+^ in roots and basal stem nodes via genes such as TaHKT1;5 (Table 1) [15,36,68].

Differences in photosynthetic pathways represent another key factor in the divergence of saline–alkali tolerance strategies. *A. thaliana*, rice, and wheat are all C3 plants; under saline–alkali stress, stomatal closure leads to CO_2_ deficiency, significantly inhibiting photosynthesis (Table 1) [89]. As a C4 plant, maize can efficiently fix low-concentration CO_2_ in mesophyll cells via phosphoenolpyruvate carboxylase (PEPC), forming C4 acids (e.g., malate). These acids are then transported to bundle sheath cells for decarboxylation, releasing concentrated CO_2_ to create a localized high-CO_2_ microenvironment around Rubisco. This mechanism directly ensures the efficient operation of the Calvin cycle even under partially closed stomatal conditions, thereby maintaining relatively stable photosynthetic efficiency [90,91]. Meanwhile, due to the CO_2_-concentrating mechanism, maize’s reliance on stomatal conductance is reduced. This means that maize requires fewer open stomata to acquire the same amount of CO_2_, significantly minimizing water loss through transpiration [92]. In saline–alkaline environments, this characteristic allows maize to utilize limited water resources more efficiently, maintain cellular turgor pressure and basic metabolic activities, and delay dehydration damage. Therefore, the C4 photosynthetic pathway not only directly safeguards energy synthesis by “maintaining CO_2_ supply,” but also optimizes the balance between “carbon acquisition and water loss.” Overall, this enhances the plant’s physiological resilience and survival capacity under stress conditions.

At the molecular regulatory level, homology and specificity coexist. Key transcription factor families identified in *A. thaliana*—such as DREB/CBF, NAC, MYB, and WRKY—are involved in the saline–alkali stress response in all three crops. For example, overexpression of *OsDREB* or *OsNAC1* significantly enhances rice tolerance [93,94]. However, the regulatory networks in crops are more complex and are linked to specific agronomic traits and developmental stages. Saline–alkali tolerance in crops like wheat and barley is closely associated with chromosomal loci such as Kna1 and Nax, which often encode HKT-type transporters, highlighting the central role of natural structural gene variation (Table 1) [95]. Breeding selection pressures have also shaped unique allelic variation pools, leading to diversification and specialization in the molecular basis of tolerance [39].

In summary, *A. thaliana*, rice, maize, and wheat share a conserved cellular and molecular “toolkit,” including mechanisms for ion homeostasis, osmotic adjustment, and antioxidant defense. The primary differences lie in higher-level physiological and structural adaptations: rice relies on a sophisticated filtration system built by its roots and stem nodes; maize benefits from the high efficiency of the C4 photosynthetic pathway and strong osmotic adjustment capacity; and both maize and wheat excel at tissue-level ion compartmentalization.

**Table 1:** Comparative analysis of saline–alkali tolerance mechanisms among *A. thaliana*, rice, maize, and wheat.

Mechanism Category	*A. thaliana*	Rice	Maize	Wheat	References
Classification	Dicotyledon, Brassicaceae.	Monocotyledon, Poaceae.	Monocotyledon, Poaceae.	Monocotyledon, Poaceae.	[96]
Root Salt Exclusion	Regulation by ion channels and transporters on the cell membrane.	Synergistic action of cortical and endodermal cells to reduce Na^+^ upward transport.	Developed root structure that effectively reduces partial Na^+^ uptake.	Root exudates and root architecture contribute to salt exclusion.	[22,85,88]
Na^+^ Selective Transport	The SOS pathway exports Na^+^ from the cell or sequesters it into the vacuole; *AtHKT1;1* mediates xylem Na^+^ unloading.	High expression of *OsHKT1;5* retrieves Na^+^ from the xylem back into the perivascular cells, reducing shootward transport.	*ZmHKT1* and its homologous genes mediate xylem Na^+^ unloading.	*TaHKT1;5*, *TaHKT2;1*, and their homologs regulate Na^+^ transport and distribution.	[7,39,52,70]
Vacuolar Sequestration	Sequesters Na^+^ into the vacuole via NHX-type Na^+^/H^+^ antiporters.	The OsNHX family, particularly *OsNHX1, OsNHX4*, and *OsNHX5*.	The *ZmNHX* family exhibits strong sequestration capacity in leaves, especially within bundle sheath cells.	The *TaNHX* family: tolerant varieties can sequester more Na^+^ in the vacuoles of mesophyll cells.	[83]
Tissue-Level Compartmentalization	Not significant.	A key mechanism; stem nodes act as crucial “filters”.	A key mechanism; capable of accumulating Na^+^ in older leaves, protecting new leaves.	A key mechanism; employs a similar “sacrifice old leaves” strategy.	[36,57,72,88]
Major Compatible Solutes	Proline, glycine betaine, soluble sugars.	Proline, glycine betaine, soluble sugars.	Proline	Proline, glycine betaine, soluble sugars.	[26]
Photosynthetic Type	C3 plant, sensitive to CO_2_ deficiency caused by stomatal closure.	C3 plant; stomatal conductance declines rapidly under salt stress, leading to significant photosynthesis inhibition.	C4 plant; maintains relatively high intercellular CO_2_ concentration and stable photosynthesis even when stomata are partially closed.	C3 plant; sensitive to stomatal limitations, but carbon assimilation stability varies among cultivars.	[14,89]
Core Signaling	ABA is the core signal, regulating a large number of stress-responsive genes.	The ABA signaling pathway is crucial, regulating stomatal closure and tolerance gene expression.	ABA signaling is core, but its interaction with hormones like JA and BR may be crop-specific.	ABA signaling is core, and its balance with hormones like cytokinin influences the senescence process.	[29,46,78]
Key Transcription Factors	*DREB/CBF*, *NAC*, *MYB*, *WRKY*, etc.	*OsDREB*, *OsNAC*, *OsbZIP*, etc.	*ZmDREB*, *ZmbZIP*, *ZmNAC*, etc.	*TaDREB*, *TaNAC*, *TaWRKY*, etc.	[31,44,48,63,94]

## 7. Perspectives

With the ongoing intensification of global climate change and cropland salinization–alkalinization, research on plant saline–alkali tolerance is progressively transitioning from fundamental mechanism exploration to a critical phase of targeted crop improvement [19]. However, current research still faces challenges, including the translational bottleneck from model systems to crop application and deeper issues such as the coordinated regulation of multiple genes and the integration of species-specific adaptation mechanisms [82]. Future studies should place greater emphasis on shifting from “gene characterization” to “system design,” constructing crop-specific saline–alkali tolerance regulatory networks by integrating multi-omics data and systems biology approaches [32]. This entails not only an in-depth investigation into the functional divergence and regulatory logic of core gene families like *SOS*, *NHX*, and *HKT* across different crop genetic backgrounds but also consideration of their complexities at the levels of tissue-specific expression, allelic variation, and post-transcriptional modifications. This will lay a solid theoretical foundation for achieving genuine “molecular design breeding” [16].

Concurrently with the deepening of mechanistic research, the precise regulation of crop-specific stress-tolerant structures will become a vital direction for future breeding. Staple food crops have developed unique saline–alkali tolerance structures through long-term evolution and artificial selection, such as the “ion filter” in rice stem nodes, the Casparian strip barrier in maize roots, and the ion retention zones in wheat basal internodes. These structures serve as crucial vehicles for crops to cope with saline–alkali stress at the whole-plant level. Utilizing gene-editing technologies to precisely regulate genes governing the development and function of these structures—for instance, enhancing the expression efficiency of *OsHKT1;5* in rice stem nodes or optimizing the *ZmSTL1*-mediated lignification degree of the Casparian strip in maize—can achieve a leap from cellular tolerance to whole-plant structural adaptation. Furthermore, as saline–alkali tolerance is a typical complex quantitative trait, its improvement urgently requires the integration and pyramiding of multi-layered tolerance mechanisms. Future breeding strategies should no longer be confined to stacking single genes or pathways but should focus on the synergistic optimization of multiple systems, including ion homeostasis, osmotic adjustment, antioxidant defense, hormone signaling networks, and photosynthetic carbon metabolism. For example, through multi-gene stacking strategies, cellular-level osmotic adjustment capacity (e.g., efficient proline synthesis) can be organically integrated with whole-plant structural adaptations (e.g., maintenance of the C4 photosynthetic system and organ-level ion compartmentalization) to develop new germplasm with broad-spectrum and durable stress resilience. This necessitates closer collaboration between breeders and molecular biologists to establish cross-scale trait integration and evaluation systems.

Moreover, fully exploiting and utilizing the natural variation harbored within crop germplasm resources is another key to breaking through the bottlenecks in saline–alkali tolerance breeding [95]. With decreasing costs of genome sequencing and advances in functional genomics technologies, resequencing core germplasm resources combined with GWAS and mutant library screening enables the efficient identification of superior alleles key to regulating ion transport, sequestration efficiency, and stress signal transduction [97]. Employing gene editing-assisted rapid domestication strategies to introduce these natural variants or artificially optimized versions into elite cultivar backgrounds will significantly accelerate the development and selection of saline–alkali tolerant varieties [98].

In recent years, gene-editing technologies such as CRISPR/Cas9 have provided revolutionary tools for precisely improving salt–alkaline tolerance within the genetic background of staple crops. Compared to traditional breeding methods, CRISPR/Cas9 technology can shorten the breeding cycle, thereby significantly reducing costs. At the same time, CRISPR/Cas9 is more accurate than conventional breeding, as it only induces mutations in target genes without altering others. For instance, using CRISPR-Cas9 to target and knock out the negative regulator *OsRR22* in rice significantly enhances the plant’s salt tolerance [99,100] and editing the drought- and salt-tolerant gene *OsDST* improves salt–alkaline tolerance in the indica rice cultivar MTU1010 [101]. In wheat, leveraging the polyploid nature of the crop, CRISPR-Cas9-mediated simultaneous editing of TaSPL6 in the A, B, and D subgenomes can alleviate its inhibition of TaHKT1;5, thereby enhancing Na^+^ retention without affecting yield-related traits [70]. In maize, precise editing of the promoter regions of *ZmSK3/ZmSK4* or *ZmHKT2* can release their suppression of the SOS pathway or optimize ion transport efficiency [51]. Moreover, with the maturation of base editing and prime editing technologies, it has become possible to achieve precise substitution of specific amino acid sites without generating DNA double-strand breaks. For example, targeted evolution of the transmembrane domains of *OsHKT1;5* or *ZmHKT1;1* can optimize their Na^+^/K^+^ transport selectivity [56]. In the future, combining genome-wide CRISPR screening with machine learning predictions holds promise for discovering more conserved or species-specific regulatory nodes for salt–alkaline tolerance, enabling an upgrade from single-gene editing to precise multi-gene regulatory networks. This approach will facilitate the development of new high-yield and stable cereal varieties adapted to marginal soils.

On the basis of systematically deciphering the molecular mechanisms of plant intrinsic saline–alkaline tolerance, harnessing beneficial rhizospheric and endophytic microorganisms to enhance plant salt resistance has emerged as a promising ecological strategy to improve crop productivity in saline–alkaline environments through microbiome research. Existing studies indicate that core microbial groups in the rhizosphere community, such as Pseudomonas, show significantly higher abundance under salt stress. Functional profiling reveals a significant enrichment of pathways related to energy metabolism, ABC transporters, amino acid biosynthesis, and ion regulation. These functional traits are directly linked to microbial adaptation to salt stress and their plant growth-promoting effects, including phosphorus solubilization, nitrogen fixation, and the synthesis of ACC deaminase and IAA. Notably, Pseudomonas aeruginosa isolated from the rhizosphere of Spartina alterniflora has demonstrated efficient phosphorus solubilization, nitrogen fixation, and high salt tolerance [102]. Utilizing beneficial rhizospheric and endophytic microorganisms as bio-inoculants to regulate the rhizosphere microenvironment, enhance nutrient uptake and stress tolerance in plants, and thereby construct a synergistic “plant-microbe” system represents a sustainable and efficient bioremediation pathway for increasing crop yield in saline–alkaline soils.

However, transitioning from laboratory breakthroughs to large-scale field applications presents multiple practical challenges for these technologies. First, the target trait is regulated by multiple factors, involving a complex quantitative genetic basis and often exhibiting genetic trade-offs with agronomic traits such as yield and quality. Second, field salinity stress is characterized by high spatiotemporal heterogeneity and is frequently intertwined with other stressors such as drought and nutrient deficiencies, necessitating crops with comprehensive stress resilience. Third, strong genotype-by-environment interactions mean that gene effects validated in controlled settings may exhibit unstable expression or diminished efficacy in the complex field environment. Additionally, regulatory policies and public acceptance of genetically modified crops constitute significant constraints for commercialization. To address these challenges, future sustainable solutions should focus on multidimensional synergistic innovations: at the strategic level, promoting a “crop-centric” systematic design breeding approach to synergistically optimize complete stress response pathways—from ion homeostasis to photosynthetic efficiency—through genomic selection and multi-gene stacking technologies; at the technical level, employing multi-candidate gene combination analyses to identify robust gene combinations that perform consistently across diverse stress scenarios; at the resource level, enhancing the exploration and utilization of hidden genetic variation in wild relatives and local landraces. Only through the integrated cycle of in-depth molecular mechanism elucidation, breakthroughs in intelligent breeding technologies, and field adaptability validation can we develop a new generation of crop varieties truly suited to saline–alkaline ecosystems and capable of achieving sustainable production.

In summary, future research on plant saline–alkali tolerance must steadfastly adhere to a “crop-centric” path, deeply integrating cutting-edge basic biology with the practical needs of breeding. Through interdisciplinary convergence and technology integration, we can gradually achieve precise design, systematic optimization, and smart breeding of saline–alkali tolerance traits. Only in this way can a solid bridge be built between the sustainable utilization of saline–alkali land resources and global food security, providing core technological support for constructing a climate-resilient modern agricultural system.

## Data Availability

No new data were created or analyzed in this study.
